# Fundamental Studies on CO_2_ Sequestration of Concrete Slurry Water Using Supercritical CO_2_

**DOI:** 10.3390/ma15010094

**Published:** 2021-12-23

**Authors:** Sang-Rak Sim, Dong-Woo Ryu

**Affiliations:** Department of Architectural Engineering, Daejin University, Pocheon-si 11159, Korea; simsr@daejin.ac.kr

**Keywords:** concrete slurry water, CO_2_ sequestration, supercritical CO_2_

## Abstract

To prevent drastic climate change due to global warming, it is necessary to transition to a carbon-neutral society by reducing greenhouse gas emissions in all industrial sectors. This study aims to prepare measures to reduce the greenhouse gas in the cement industry, which is a large source of greenhouse gas emissions. The research uses supercritical CO_2_ carbonation to develop a carbon utilization fixation technology that uses concrete slurry water generated via concrete production as a new CO_2_ fixation source. Experiments were conducted using this concrete slurry water and supernatant water under different conditions of temperature (40 and 80 °C), pressure (100 and 150 bar), and reaction time (10 and 30 min). The results showed that reaction for 10 min was sufficient for complete carbonation at a sludge solids content of 5%. However, reaction products of supernatant water could not be identified due to the presence of Ca(HCO_3_)_2_ as an aqueous solution, warranting further research.

## 1. Introduction

Concrete is the most widely used construction material, and approximately 4100 million metric tons of concrete were produced globally in 2020 [1,2,3]. According to a United Nations (UN) report, the current world population is predicted to increase from 7.7 billion to 9.7 billion by 2050, i.e., an increase of nearly 2 billion [4]. Such population growth is expected to increase the demand for social infrastructure facilities and housing, especially in developing countries, which will lead to a sharp increase in concrete consumption [5,6].

Cement, the main binder material of concrete, is produced by mixing and pulverizing limestone (primary raw material; CaCO_3_) with other clay minerals (SiO_2_, Al_2_O_3_, Fe_2_O_3_, etc.) and sintering at a high temperature above 1450 °C. The amount of CO_2_ generated during sintering accounts for approximately 93% of the total emissions during cement manufacturing, where 33% originates from the combustion of fossil fuels and 60% from the decomposition of limestone. The process of limestone decomposition proceeds according to Equation (1) [7,8,9].

CaCO_3_ + heat → CaO + CO_2_ (1)

**Figure 1:**
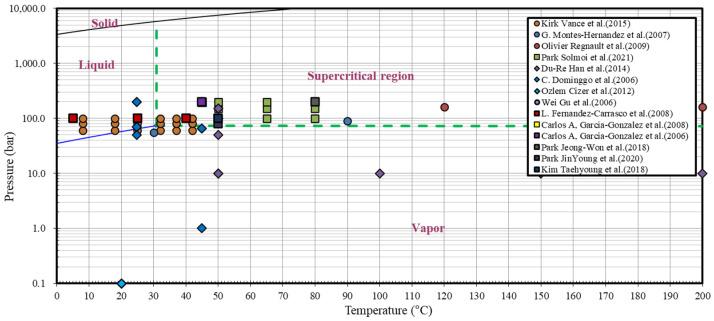
Temperature–pressure conditions reported in the current literature on supercritical CO_2_ mineral carbonation.

Various other industries are also responsible for emitting a significant amount of greenhouse gases, and such increase in greenhouse gas emissions leads to intensified global warming and rapid climate change [10]. Carbon capture and sequestration (CCS) technology has been developed for capturing CO_2_ emitted from industrial sectors and sequestering it in the Earth’s strata to maintain environmental sustainability by preventing the average global temperature from reaching 1.5 °C above pre-industrial levels. However, problems such as leakage of CO_2_ isolated in the stratum and groundwater pollution are recognized as concerns associated with CCS [11]. Therefore, extensive research has been directed to developing carbon capture and utilization (CCU) technology based on mineral carbonation, which chemically bonds the captured CO_2_ as carbonate minerals and utilizes it as an effective resource [12]. Since typical conversion of CO_2_ to carbonate is very slow and inefficient under the conditions of ambient temperature and pressure, there has recently been an increasing trend in research on maximizing the mineral carbonation reaction using supercritical CO_2_. Existing studies on mineral carbonation using supercritical CO_2_ have revealed an absence of any significant effect of temperature in the supercritical state and have also shown that the carbonation efficiency increases with increasing pressure [12,13,14,15,16,17,18,19,20,21]. The temperature–pressure conditions explored in recent studies on mineral carbonation using supercritical CO_2_ are summarized in Figure 1.

During concrete production, the generation of concrete slurry water is inevitable. Concrete slurry water refers to the surplus concrete returned from over orders or from failure to meet the requirements of construction sites and concrete cleaning requirements, which ensure that concrete does not adhere to truck agitators or mixers in batching plants at ready-mixed concrete factories. Since concrete slurry waste is strongly alkaline, it may contaminate the soil and water and, thus, needs to be neutralized or treated with recycling equipment, such as a filter press [22,23,24].

In general, concrete slurry waste is a dehydrated cake primarily obtained from air drying or filter pressing concrete slurry water upon separation into residual aggregate and coarse aggregate in a ready-mixed concrete factory. The waste contains a large amount of Ca^2+^ derived from the non-hydrated cement component; therefore, it shows promise for application as a material for immobilizing a substantial amount of CO_2_ [25,26].

However, most reported studies on concrete slurry waste have primarily explored the use of a small amount of dry/wet concrete slurry waste as a cement substitute, and there is currently a lack of reported research on the use of concrete slurry waste for large-scale CO_2_ capture.

In addition, for supernatant water that lies above the precipitate of concrete slurry water, hydration is delayed by 0.5–2 h or more due to the coating of cement gel formed around the non-hydrated cement. It is understood that the Ca^2+^ concentration increases in the supernatant water during this induction period and that CO_2_ may react with the ions to be sequestered in the form of CaCO_3_ [27].

Therefore, in this study, we investigate the possibility of large-scale CO_2_ sequestration by concrete slurry water and supernatant water by utilizing a supercritical CO_2_ reaction and exploring the effects of temperature (40 and 80 °C), pressure (100 and 150 bar), and reaction time (10 and 30 min).

## 2. Experimental

### 2.1. Materials

**Figure 2:**
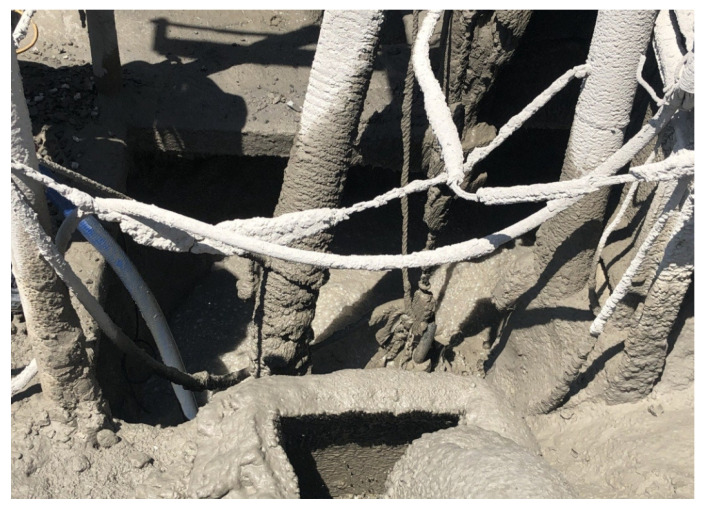
Concrete slurry water extraction.

Concrete slurry water was obtained from a ready-mixed concrete factory owned by company “Y”, located in Gyeonggi-do, South Korea. The concrete slurry water extraction is illustrated in Figure 2. The concrete slurry water used in experiments was separated into supernatant water and concrete slurry waste to obtain equal sludge solids content. The concrete slurry waste was dried at 105 °C until it reached a constant weight. Then, the dried concrete slurry waste was pulverized to control the particle size to 75 µm or below using a 200 mesh. Mineral carbonation using a supercritical CO_2_ reaction was initiated by diluting 2 kg of supernatant water and 100 g of dried concrete slurry waste. The chemical compositions of the supernatant water and concrete slurry waste are presented in Table 1 and Table 2, respectively.

### 2.2. Supercritical CO_2_ Reactor

**Figure 3:**
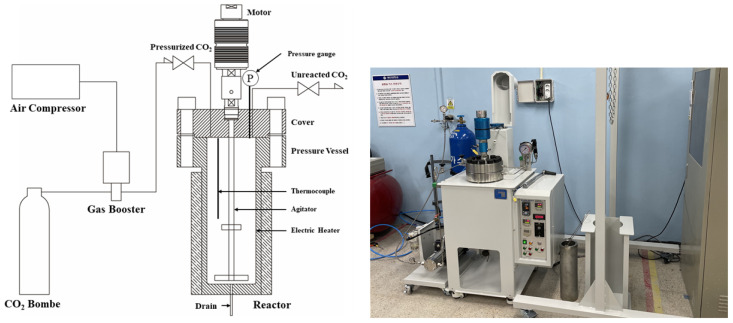
Schematic and photograph of the supercritical CO_2_ reactor.

Figure 3 shows a schematic representation of the supercritical CO_2_ reactor, comprising a gas booster (Maximator, Nordhausen, Germany) and a reactor (PHOS-ENTECH, Seoul, Korea). The reactor is equipped with an electrical heater for temperature control, an agitator for mixing, and a thermocouple and a pressure gauge for temperature and pressure measurements, respectively. The gas booster is connected to an air compressor that maintains the CO_2_ in its supercritical state by pressurizing the reactor vessel with CO_2_ gas at high pressure.

The maximum operating temperature and pressure of the supercritical CO_2_ reactor were 80 °C and 200 bar, respectively, and the internal volume of the reactor was 4 L. In addition, the agitator was designed to enable control of the rotation speed up to 400 rpm.

### 2.3. Supercritical CO_2_ Carbonation

**Figure 4:**
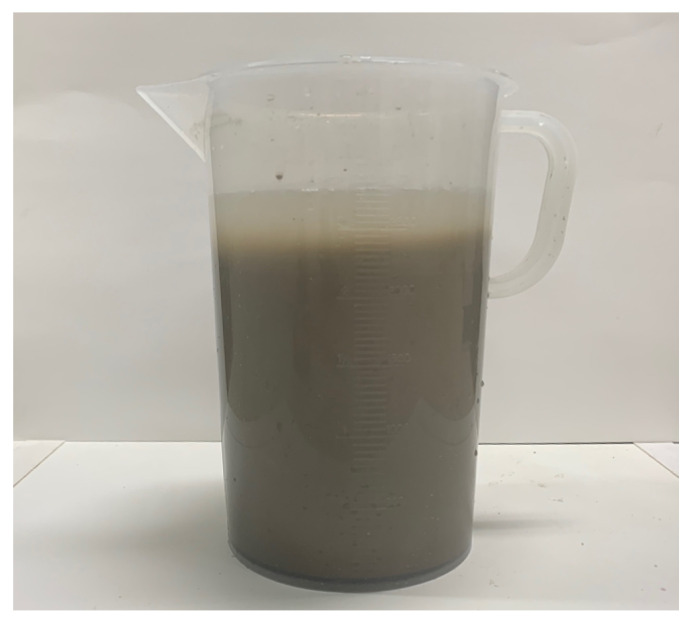
Concrete slurry water.

Carbonation experiments using supercritical CO_2_ were performed using the following algorithm:

- The sample diluted with supernatant water and concrete slurry waste is added to the reactor, which is then assembled. The concrete slurry water used in this study is shown in Figure 4.
- The electric heater is powered on. Once the reactor vessel reaches the target temperature, CO_2_ is injected until the desired pressure is achieved.
- Once the CO_2_ inside the vessel reaches the target pressure, the agitator is operated at 200 rpm to perform accelerated carbonation over the specified reaction time, while maintaining the temperature and pressure.
- After the specified reaction time has elapsed, CO_2_ is released, the reactor is disassembled, and the sample is retrieved.
- The supernatant water and concrete slurry waste are separated from the sample. The concrete slurry waste is dried at 105 °C until it reaches a constant weight.
- SEM (Philips XL30 ESEM, Eindhoven, The Netherlands), XRD (Rigaku D/max 2200+ Ultima, Tokyo, Japan), and TG–DTA (Hitachi STA 7300, Tokyo, Japan) characterizations and pH (Hanna Instruments HI2215, Woonsocket, RI, USA) measurements are conducted on the dehydrated concrete slurry waste to determine the degree of carbonation reaction.

### 2.4. Chemical Analysis

To investigate the mineralogical phase transition of the concrete slurry waste upon carbonation by supercritical CO_2_, SEM, XRD, and TG–DTA analyses and pH measurements were performed on samples before and after the carbonation reaction. The pH measurements were conducted on the eluate after mixing the sample with distilled water at a ratio of 1:5. To quantify the amount of CaCO_3_ produced from the reaction, TG–DTA measurements were performed at a ramp rate of 10 °C/min over a temperature range from 25 to 1000 °C.

## 3. Results

### 3.1. PH Measurement

**Figure 5:**
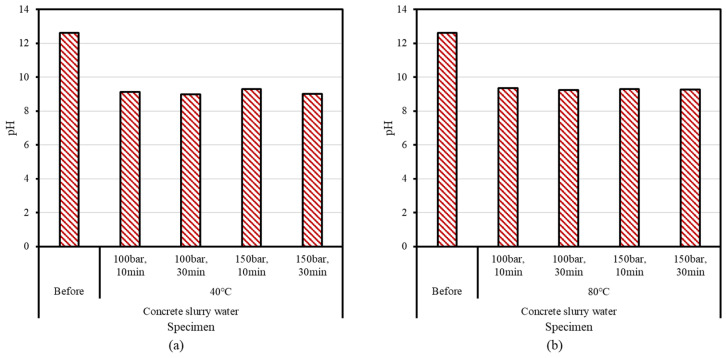
Results of pH measurements for supercritical CO_2_ carbonation at (**a**) 40 °C or (**b**) 80 °C.

The pH measurement results before and after supercritical CO_2_ carbonation are shown in Figure 5. Prior to reaction, the supernatant water and concrete slurry waste had a pH above 12, while after reaction the pH was significantly lower (9.0–9.3). Moreover, pH measurements on supercritical CO_2_ carbonation revealed no temperature and pressure dependence, but the pH was shown to decrease by 2% as the reaction time increased from 10 to 30 min. In general, the pH of high-purity CaCO_3_ is 9.4; therefore, the pH of the products of the supercritical CO_2_ carbonation reaction (9.0–9.3) indicate the conversion of CO_2_ to CaCO_3_ via the reaction shown in Equation (2).

CaO + CO_2_ → CaCO_3_ (2)

In contrast, no products were formed from supernatant water and pH measurements could not be performed. As shown in Equation (3), CaCO_3_ converts to Ca(HCO_3_)_2_ when CO_2_ is supplied continuously. In generally Ca(HCO_3_)_2_ exists as an aqueous solution. It is understood that no products were formed due to the presence of Ca(HCO_3_)_2_ in its aqueous solution state because an excessive amount of CO_2_ is available under supercritical CO_2_ conditions of supernatant water that contains a small amount of Ca^2+^ (800 mg/L). However, since the reaction shown in Equation (2) is reversible, Ca(HCO_3_)_2_ reverts to CaCO_3_ at room temperature, along with release of CO_2_ gas, and further research is required to clarify this point [28,29].

CaCO_3_(s) + CO_2_(g) + H_2_O → Ca(HCO_3_)_2_(aq) (3)

### 3.2. SEM

**Figure 6:**
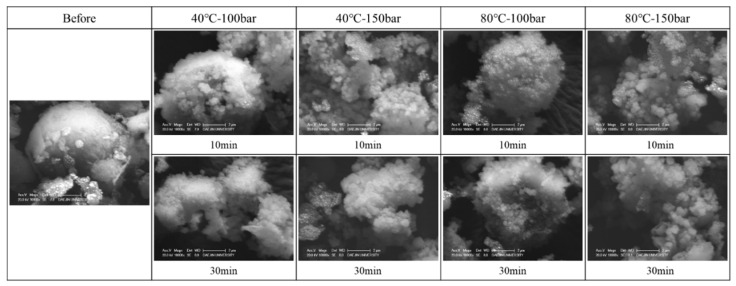
SEM characterization results.

Figure 6 shows the SEM characterization results of concrete slurry waste produced during supercritical CO_2_ carbonation. In general, in the case of Ca(OH)_2_, a microcrystalline layer of CaCO_3_ is densely formed on the surface of the particle during the carbonation reaction with supercritical CO_2_ and diffuses inwards to approach complete carbonation as the reaction proceeds [13]. In this study, particles of the concrete slurry waste before reaction showed spherical morphology without any crystal layers on the surface; however, after supercritical CO_2_ carbonation, this morphology changed to microcrystalline layers on the surface of the particle regardless of temperature, pressure, and reaction time, indicating the progression of carbonation by supercritical CO_2_.

### 3.3. XRD Measurement Results

**Figure 7:**
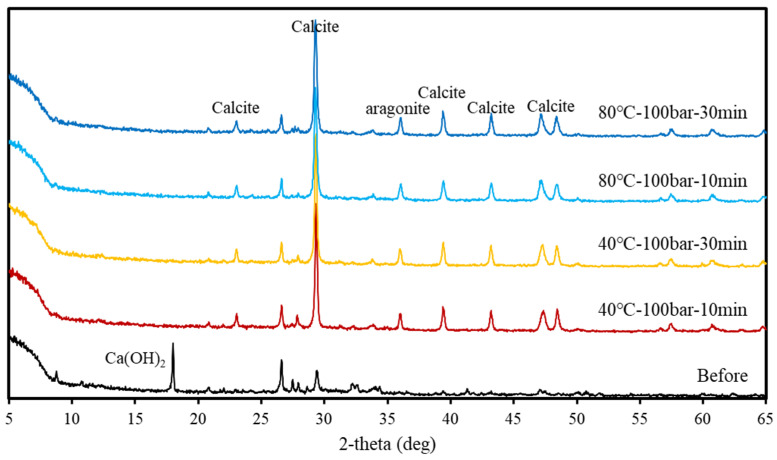
XRD patterns obtained at 100 bar.

**Figure 8:**
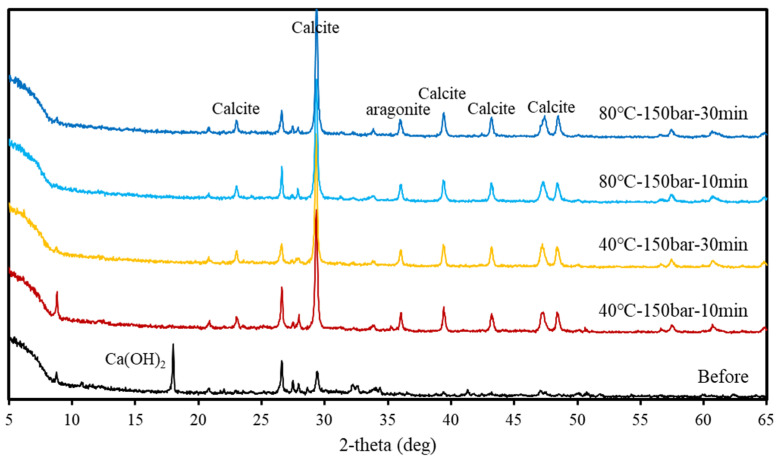
XRD patterns obtained at 150 bar.

XRD patterns obtained before and after supercritical CO_2_ carbonation are displayed in Figure 7 and Figure 8, respectively. Peaks indicating the presence of Ca(OH)_2_ and a small amount of calcite were found in the XRD pattern of concrete slurry waste before reaction; after reaction, the Ca(OH)_2_ peak disappeared, while peaks reflecting the presence of calcite and a small amount of aragonite were detected. Further, comparing the XRD patterns of the reaction products obtained at different reaction temperatures, pressures, and times showed that similar peaks appeared irrespective of the reaction conditions. The three crystal structures of CaCO_3_ are aragonite, vaterite, and calcite, among which calcite is the most stable form [21,30]. According to reported studies, calcite is mainly formed when Ca^2+^/CO_3_^2−^ ≤ 1. The supercritical CO_2_ conditions in this study also produce a large amount of CO_3_^2−^ and, therefore, a decrease in Ca^2+^/CO_3_^2−^ ratio indicates that calcite is the main reaction product [16].

### 3.4. TG–DTA Measurement Results

**Figure 9:**
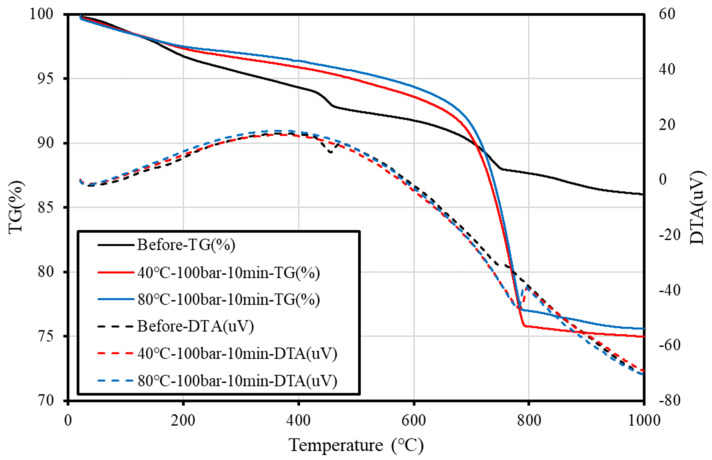
TG-DTA results obtained before and after supercritical CO_2_ carbonation.

Figure 9 and Table 3 present the TG-DTA results obtained before and after supercritical CO_2_ carbonation. Generally, the major cement hydrate, Ca(OH)_2_, undergoes pyrolysis near 450–550 °C and reacts with CO_2_ to form CaCO_3_, which pyrolyzes at 600–800 °C. The TG–DTA measurement on the sample before supercritical CO_2_ carbonation revealed small weight losses for Ca(OH)_2_ and CaCO_3_. Measurements after the reaction showed no signs of Ca(OH)_2_ weight loss, but showed signs of CaCO_3_ weight loss regardless of the temperature, pressure, and reaction time. Therefore, it was confirmed that the sludge with a solids content of 5% underwent complete carbonation in only 10 min via supercritical CO_2_ carbonation.

## 4. Conclusions

In this study, supercritical CO_2_ reactions were shown to reduce CO_2_ emissions when utilized in the developed carbon capture and utilization technology using concrete slurry water produced during concrete manufacturing as a new means for CO_2_ sequestration. The major findings are as follows:

- For supernatant water under supercritical CO_2_ conditions, reaction products could not be confirmed due to Ca(HCO_3_)_2_ existing as an aqueous solution in the presence of an excess amount of injected CO_2_. However, since the reaction is reversible, CaCO_3_ can precipitate from aqueous Ca(HCO_3_)_2_ over time at room temperature, along with the release of CO_2_ gas; hence, further research is necessary.
- Mineral carbonation of concrete slurry water by supercritical CO_2_ demonstrated that complete carbonation can be achieved in only 10 min of reaction at a sludge solids content of 5%. However, the reaction seemed to be independent of the supercritical CO_2_ temperature and pressure at 5% sludge solids content. Further investigation on the change in the reaction with respect to sludge solids content is to be performed.
- In future studies, quantitative analyses on supercritical CO_2_ carbonation (optimal temperature, pressure, and reaction time) and CO_2_ storage capacity with respect to sludge solids content will be conducted.
- Collating the results, it is possible to fix CO_2_ via supercritical CO_2_ carbonation, and the implementation of a new CO_2_ fixation source with concrete slurry water is considered viable. The implementation of supercritical CO_2_ mineral carbonation technology using concrete slurry water will enable carbon-neutrality to be achieved by reducing greenhouse gas emissions from not only cement industries, but all industrial sectors.

## Figures and Tables

**Table 1:** Chemical composition of supernatant water (obtained by ICP spectroscopy).

Chemical Composition (mg/L)				
Ca	Mg	Na	Fe	K
812	0	242	0	711

**Table 2:** Chemical composition of concrete slurry waste (obtained by XRF spectroscopy).

Chemical Composition (wt.%)							
CaO	SiO_2_	Al_2_O_3_	SO_3_	MgO	Fe_2_O_3_	K_2_O	TiO_2_
29.69	23.82	5.15	2.11	1.94	2.58	0.93	0.39

**Table 3:** The amount of Ca(OH)_2_ and CaCO_3_ before and after supercritical CO_2_ carbonation.

Specimens	Amount of Ca(OH)_2_ (%)	Amount of CaCO_3_ (%)
Before	1.11	2.79
40 °C-100 bar-10 min	0	15.94
80 °C-100 bar-10 min	0	15.55

## Data Availability

The data presented in this study are available upon request from the corresponding author.
